# The Therapeutic Outlook in Pediatric Medicine

**Published:** 1906-02-03

**Authors:** 


### Hospital Clinics.

### THE THERAPEUTIC OUTLOOK IN PEDIATRIC MEDICINE.

What Dr. Emmett Holt says upon the subject to which he has devoted his life is generally worth listening to. This is certainly the case with a discourse upon the above subject appearing recently in New York.[1] Dr. Holt observes in preface that not many years ago the discovery of certain specifics, such, for instance, as the antitoxin in the case of diphtheria, and thyroid therapy in the case of cretinism, stimulated a hopeful anticipation of an era of specifics. This hope has not been justified by events, though immense progress has been made in the department of specific medication for microbic diseases. It has become apparent that medicine is not yet the exact science that some optimists began to hope it might become, and that short cuts are likely to remain the exception in matters medical. Dr. Holt considers that the best results in the treatment of a large majority of diseases will continue to be won by a patient and intelligent application of "our better understanding of physiology and hygiene, and our newly-gained knowledge of physiological chemistry." The writer observes that the old idea that disease could be cured by drugs has disappeared, and complains that in its place has arisen a pessimistic and sceptical creed with which he has no sympathy. This is to the effect that unless a disease is amenable to surgery, treatment amounts to very little. From such a sterilising belief Dr. Holt altogether dissociates himself, affirming that the success or failure of purely medical maladies is in a very large measure dictated by the management their victims receive.

In the case of ailing children correct treatment involves a proper appreciation and application of two elements of success which, in view of their fundamental importance, are far too little comprehended by the profession at large. These are dietetics, and general hygiene. In the cases of all growing children, whether sick or well, the bodily nutrition is a matter of the very first concern, and good nutrition is only a synonym for correctness of diet and of hygiene. Consequently these two matters constitute the points of special emphasis in the treatment of disease in childhood.

Touching the matter of dietetics Dr. Holt observes that it is as idle to expect grapes from thistles as healthy children reared in defiance of the rules of physiology on foods lacking the qualities essential for normal nutrition like most of the proprietary foods on the market. Disorders of digestion in early life are very clearly due to a faulty dietary, for it is fair to assume that the majority of children are born with a healthy digestive mechanism. Therefore such disorders of digestion must be treated by dieting, and dieting must be held to be the *sine qua non* of success, and so applied. In the large bulk of cases of intestinal disorder in babyhood, whether the patients be fed at the breast or artificially, the root-evil is to be found in the diet alone. Some item of the daily repast, whether by excess or deficiency, is disturbing the normal chemistry of digestion, and the obvious indication is to identify the fault.

As illustrating this point the author quotes the case of an infant at the breast who after thriving well for a few months began to be troubled with symptoms of intestinal disturbance. The stools became loose, their colour changed from yellow to green, and they ultimately attained a frequency varying from six to eight in the course of the day. The infant's grandfather, a surgeon of high repute, observing the state of the stools, remarked that he did not wish to dictate, but that in his time a baby with such stools was dosed with calomel. This suggestion was not acted upon, but an analysis of the mother's milk established the fact that the breast milk contained a proportion of fat amounting to no less than 7 per cent. Such a result established the cause of the trouble, for the highly fatty milk clearly acted as a purge, and hurried all the intestinal contents along their course far too quickly. In this instance attention to the diet of the mother, and partial bottle-feeding with a milk containing a low percentage of fat resulted in a complete cure and a final return to breast-feeding alone. It is obvious that in such a case drug treatment is worse than useless.

Another instance quoted to point the same moral was that of an infant successfully nursed for five months. At this time a sister of the nursing mother became seriously ill, and remained in a hopeless condition. It then became apparent that the health of the infant was suffering through the anxieties of the mother. The gain in weight instead of amounting to some six or eight ounces a week, fell to one or two, while the stools became frequent and curdy. At times the child appeared to suffer from colic, and a good deal of mucus appeared in the dejections. Although in this case no actual analysis of the breast milk confirmed the suspicion, the treatment was founded on the assumption that an excessive proteid content in the milk had produced an intestinal catarrh. Consequently breast-feeding was intermitted altogether for two days, as an experiment, the diet during this interval being a weak cow's-milk containing 2 per cent. of fat, 6 per cent. of sugar, and .75 per cent. of proteid. A marked improvement followed the adoption of this *régime*. Presently an attempt was made to return to breast-feeding by the alternate use of the breast and the bottle, but even this modification of breast-feeding led to a return of the catarrhal symptoms. In consequence it was considered wise to give up the administration of the breast entirely, in spite of the fact that the mother's supply was ample. A purely artificial diet resulted in a complete cure in three days. In such a case, again, the employment of drugs, diarrhœa-mixtures, and such things, is obviously futile, even when it is not actually harmful.

As in the case of chronic indigestion so in acute varieties, attention to diet is the only road to successful treatment; but whereas in the chronic disorders the fault will commonly be found in an excess of one particular element, in acute lesions of the intestinal tract all varieties of food are an offence. The sovereign remedy for all acute dyspepsias is starvation for a time, this to be followed by a very cautious and gradual return to a normal dietary. It is pointed out that of the three chief chemical classes of food, carbohydrates are the easiest of digestion, and should therefore be the first to be re-admitted after the starvation-time of treatment. Fats are contra-indicated except in very reduced quantity; proteids also must be given with a sparing hand. The casein of milk should therefore be removed by curdling, and the whey alone administered for a time. Dr. Holt insists upon the importance of giving an ample allowance of water to such patients as those we are now considering. If water be given in abundance, starvation is borne with extraordinarily little disturbance. Some children will not take water by itself, but will take it readily when disguised in the form of a very weak food, or when flavoured with a little sugar and lemon juice. In these circumstances, when the intake of nourishment is reduced to a minimum, it is, of course, necessary to reduce as far as possible all drafts upon the energy of the child. Complete rest is therefore essential. The treatment of acute intestinal diseases in childhood is summed up as follows: Evacuants, diet, rest, and water. In the case of chronic indigestion, diet is, as has been said, the only remedy, and care must be exercised for a long period when the ailment is of long standing. It is a common error to regard the wasted condition of some patients of this class as an indication for "feeding up": a full diet is therefore prescribed, which is for the time being quite unassimilable and merely perpetuates the disorder. The necessity for prolonged treatment and a cautious relaxation of embargoes upon a generous diet is evidenced by a child who, having after three months of careful treatment begun to make headway, returned to her home, was seen by the family physician, and considered to be a suitable for "feeding up." She was therefore freely indulged in cream, potatoes, rice, and bread (all the articles, in fact, which had been rigorously excluded), relapsed almost immediately, and nearly lost her life. In another case under a generous régime an analysis proved the fæces to consist of fat to the extent of 65 per cent.

Dr. Holt regards a large majority of the common neuroses of childhood as directly dependent upon erroneous dieting and hygiene. Chorea, for instance, insomnia, night-terrors, habit-spasm, convulsions, hysteria, enuresis and neurasthenia. These conditions, he considers, are eminently unsuitable for drug-treatment. Many a child affected with chorea, he affirms, is dosed freely with arsenic, whose ailment would be far more suitably met by sending her to bed at seven o'clock instead of ten, by denying her tea and coffee, by keeping her from school even at the expense of a much-prized and expected scholarship, and by a careful regulation of the hours of recreation and exercise.

Speaking again of convulsions occurring once or twice a year, the author says: "Few of these children have epilepsy at the time, or subsequently develop it. In the great majority of cases the attacks of convulsions are due to digestive disorders and consequent disturbances of nutrition. When once a proper diet has been carried into effect, the chronic constipation overcome, and a suitable régime inaugurated, we regularly see these attacks lessening in frequency and severity, and, in a great majority, ultimately disappear."

The general drift of Dr. Emmett Holt's teaching may be gathered from the following concluding paragraphs. "Let us get rid of the notion that because the child is ill he requires medicine if he is to get well; and above all let us not get into the habit of treating the thermometer, nor even the name of the disease." "Our greatest need to-day, I believe, is a more scientific and intelligent knowledge of practical dietetics, and a better understanding of the conditions of health and growth. We must realise that in every acute disease and chronic disorder it is of the first importance that we should have a knowledge of how best to preserve the nutrition of the body."

[1] Med. News, Dec. 23, 1905.

